# Genetic dissection of major QTL for grain number per spike on chromosomes 5A and 6A in bread wheat (*Triticum aestivum* L.)

**DOI:** 10.3389/fpls.2023.1305547

**Published:** 2024-01-08

**Authors:** Cheng Jiang, Zhibin Xu, Xiaoli Fan, Qiang Zhou, Guangsi Ji, Simin Liao, Yanlin Wang, Fang Ma, Yun Zhao, Tao Wang, Bo Feng

**Affiliations:** ^1^ Chengdu Institute of Biology, Chinese Academy of Sciences, Chengdu, China; ^2^ College of Life Sciences, Sichuan University, Chengdu, China; ^3^ University of Chinese Academy of Sciences, Beijing, China; ^4^ The Innovative of Seed Design, Chinese Academy of Sciences, Beijing, China

**Keywords:** QTL mapping, BSE-Seq, grain number per spike, haplotype analysis, wheat

## Abstract

Grain number per spike (GNS) is a crucial component of grain yield and plays a significant role in improving wheat yield. To identify quantitative trait loci (QTL) associated with GNS, a recombinant inbred line (RIL) population derived from the cross of Zhongkemai 13F10 and Chuanmai 42 was employed to conduct QTL mapping across eight environments. Based on the bulked segregant exome sequencing (BSE-Seq), genomic regions associated with GNS were detected on chromosomes 5A and 6A. According to the constructed genetic maps, two major QTL *QGns.cib-5A* (LOD = 4.35–8.16, PVE = 8.46–14.43%) and *QGns.cib-6A* (LOD = 3.82–30.80, PVE = 5.44–12.38%) were detected in five and four environments, respectively. *QGns.cib-6A* is a QTL cluster for other seven yield-related traits. *QGns.cib-5A* and *QGns.cib-6A* were further validated using linked Kompetitive Allele Specific PCR (KASP) markers in different genetic backgrounds. *QGns.cib-5A* exhibited pleiotropic effects on productive tiller number (PTN), spike length (SL), fertile spikelet number per spike (FSN), and ratio of grain length to grain width (GL/GW) but did not significantly affect thousand grain weight (TGW). Haplotype analysis revealed that *QGns.cib-5A* and *QGns.cib-6A* were the targets of artificial selection during wheat improvement. Candidate genes for *QGns.cib-5A* and *QGns.cib-6A* were predicted by analyzing gene annotation, spatiotemporal expression patterns, and orthologous and sequence differences. These findings will be valuable for fine mapping and map-based cloning of genes underlying *QGns.cib-5A* and *QGns.cib-6A*.

## Introduction

Wheat (*Triticum aestivum* L.) is a vital crop that provides a substantial portion of the world’s food. However, as the world population continues to grow, the demand for food is increasing. Despite the current annual growth rate of wheat production reaching 0.9%, it falls short of the required annual growth rate of approximately 2.4% needed to sustain the world population by 2050 (Ray et al., 2013; Gao, 2021). As a result, enhancing the yield potential has become a fundamental objective in wheat breeding. Grain yield is a complex quantitative trait mainly determined by three factors: spike number per unit area, thousand grain weight (TGW), and grain number per spike (GNS). Therefore, revealing the genetic factors underlying GNS is essential to improve yield potential.

The genetic regulatory pathways governing architecture of the inflorescence play a crucial role in determining GNS in wheat. Generally, spike development can be divided into three main phases: the duration of the flowering transition; initiation, distribution, and termination of spikelet meristem (SM); formation and generation of floret meristem (FM) (Luo et al., 2023). During the flowering transition period, several widely recognized genes involved in flowering time participate in regulating the timing of inflorescence meristem (IM) differentiation and the initiation of spikelet and floret development. During the vernalization-induced flowering process, *VERNALIZATION 1* (*VRN1*) serves as a central regulatory gene, playing a crucial role in maintaining IM activity and controlling SM characteristics. Similar to VRN1, FRUITFULL2 (FUL2) and FRUITFULL3 (FUL3) redundantly facilitate the transition from SAM to IM (Li et al., 2019). The photoperiod gene *Photoperiod-1* (*Ppd-1*) in wheat influences inflorescence structure. Insufficient Ppd-D1 leads to the formation of paired spikelet and an increase in grain count under short sunlight conditions (Boden et al., 2015). VRN3/TaFT1, a homolog of Flowering Locus T (FT) in *Arabidopsis* and the Heading date 3a (Hd3a) in rice, interacts with the transcription factor TaFDL2 to activate *VRN1* (Yan et al., 2006; Li and Dubcovsky, 2008). Additionally, VRN1 and Ppd-1 positively regulate *VRN3*/*TaFT1* and *TaFT2*, whereas VRN2 is known to act as a transcriptional inhibitor of these genes (Yan et al., 2004; Chen and Dubcovsky, 2012; Shaw et al., 2019). TaFT2 controls the initiation and quantification of spikelets. *PHYTOCHROME C* (*PHYC*) acts as an upstream regulatory factor, activating *Ppd-1* and *VRN3*/*TaFT1*. The TCP transcription factor TEOSINTE BRANCHED 1 (TaTB1) inhibits spike formation (Dixon et al., 2018).

The transition from IM to SM is crucial for establishing the inflorescence structure in Gramineae plants during spikelet initiation, distribution, and termination. Overexpression of wheat *AGAMOUS-LIKE6* (*TaAGL6*) affects the expression of meristem-active genes like *FUL2* and *TaMADS55*, resulting in a significant increase in the number of spikelets and grains per spike (Kong et al., 2021). Wheat FRIZY PANICLE (WFZP), a member of the class II AP2/ERF transcription factor (TF), directly activates *VRN1-A* and *HOMEOBOX4* (*TaHOX4*)-*A*. In addition, *WFZP* also acts as an inhibitor of the spikelet formation gene *BARREN STALK1* (*TaBA1*), exerting a dual effect (Poursarebani et al., 2015; Du et al., 2021; Li et al., 2021). The microRNA156 (miR156)-SPL module is crucial in initiating SM development during wheat spike development. MiR156 regulates SPL family genes, including *TaSPL3*/*17* in wheat. TaSPL3/17 interacts with DWARF53 (TaD53) to regulate the expression of genes *TaTB1* and *TaBA1*, which are involved in the differentiation of spikelet meristem and floral meristem. This interaction ultimately affects wheat spikelet development (Liu et al., 2017). The miR172-AP2 module also plays a critical role in the proper development of spikelets (Debernardi et al., 2017; Zhong et al., 2021). The *q*/*ap2l5* mutant exhibits a significant reduction in spikelet number, which can be attributed to the premature transformation of spikelet meristem into terminal spikelet (Debernardi et al., 2020).

The interactions among MADS, SPL, TCP, and AP2 TFs play an essential role in promoting or maintaining the characteristics of SM and FM, which significantly influence the development of wheat floret. The E-class *SEP* genes are primarily responsible for regulating the floral organs’ development (Pelaz et al., 2000; Ditta et al., 2004). Upregulation *TaVRT2*, a MADS-box gene belonging to the *SHORT VEGETATIVE PHASE* (*SVP*) branch, causes the downregulation of *TaSEP1*. As a result, the transformation of spikelet into floret is delayed, resulting in an increased number of basal spikelets (Backhaus et al., 2022). The SQUAMOSA proteins VRN1 and FUL2 function as repressors of the *SVP* branch MADS box genes, such as *TaVRT2*, *TaSVP1*, and *TaSVP3*. These proteins stimulate the formation of small flowers following the transition to flowering (Li et al., 2019; Li et al., 2021; Liu et al., 2021). Therefore, the downregulation of SQUAMOSA protein for *SVP* gene expression is essential to promoting *SEP* gene expression and ensuring normal flower development (Li et al., 2021; Backhaus et al., 2022). Upregulation of the miR156 target gene *TaSPL13* leads to an increased production of small flowers and grains per spike in wheat (Li et al., 2020). *Q*/*AP2L5* and *AP2L2* redundantly recognize and prevent small flowers from degenerating into glumes through miR172-guided mechanisms (Debernardi et al., 2017; Debernardi et al., 2020).

Like other traits related to yield, GNS is a quantitative trait influenced by both genetic and environmental factors. As a result, researchers have preliminarily focused on mapping quantitative trait loci (QTL) in various genetic or natural populations of wheat. Up to now, numerous QTL associated with GNS have been identified across 21 chromosomes in previous studies (Börner et al., 2002; Peng et al., 2003; Huang et al., 2004; Quarrie et al., 2005; Liu et al., 2006; Kumar et al., 2007; Cuthbert et al., 2008; Wang et al., 2009; McIntyre et al., 2010; Wang et al., 2011; Blanco et al., 2012; Rustgi et al., 2013; Azadi et al., 2014; Gao et al., 2015; Zhang et al., 2016; Roncallo et al., 2017; Guan et al., 2018; Liu et al., 2019; Hu et al., 2020; Mizuno et al., 2021; Qiao et al., 2022; Hu et al., 2023). However, few major QTL have been found that can be detected in multiple environments and validated in different genetic backgrounds, hindering their utilization in breeding programs. Therefore, it is essential to identify and validate the novel QTL/genes associated with GNS.

In the present study, we utilized bulked segregant exome sequencing (BSE-Seq) and linkage analysis to identify QTL that control GNS. The major QTL were subsequently validated in different genetic backgrounds, and potential candidate genes were predicted. Additionally, an analysis of the haplotypes of the major QTL was conducted.

## Materials and methods

### Plant materials and field trials

Three genetic populations obtained through the single-seed descent method as well as a natural population were employed in this study. They were (1) a recombinant inbred line (RIL) population (13CM, 316 F_7_ lines) derived from the cross of Zhongkemai 13F10 (ZKM13F10) and Chuanmai 42 (CM42); (2) an F_2_ population (CZ5782, 184 individuals) derived from the cross of Chuanmai 104 (CM104) and ZM5782; (3) an F_2_ population (CS352, 126 individuals) derived from the cross of CM104 and SH352; and (4) a natural population containing 321 wheat accessions, including 59 widely grown cultivars during the last two decades and 262 accessions of Chinese wheat mini-core collection (88 modern cultivars, 17 introduced cultivars, and 157 landraces) (Li et al., 2022). The 13CM population was used to construct genetic map and detect QTL; CZ5782 and CS352 were used to validate the target QTL in different genetic backgrounds, and the natural population was used for haplotype analysis.

ZKM13F10 (ZKM138/PW18) is a stable breeding line selected by our lab characterized by high GNS. CM42 (Syncd768/SW3243//Chuan6415) is a core cultivar that has been used as one of the parents to develop more than 50 new cultivars in China. It possesses desirable yield-related traits including high grain weight and wide adaptability. CM104 is a cultivar derived from CM42 and inherits its major elite traits (including high grain weight and long spike). SH352 and ZM5782 are wheat lines to construct populations used for validating the major QTL.

The 13CM population and its parents were cultivated in eight different environments: Shuangliu (103°52′E, 30°34′N) during the 2017–2018, 2018–2019, 2019–2020, and 2020–2021 growing seasons (referred to as E1, E3, E5, and E7, respectively), and Shifang (104°11′E, 31°6′N) during the 2017–2018, 2018–2019, 2019–2020, and 2020–2021 seasons (referred to as E2, E4, E6, and E8, respectively). CZ5782 and CS352 individuals were cultivated in Shifang during the 2021–2022 growing season. Each plot had two rows. The row length and row spacing were 1.2 m and 0.2 m, respectively, and each row sowed 12 seeds. At sowing time, the fertilizer (N: 25%, P_2_O_5_: 10%, K_2_O: 10%) was applied with 450 kg/ha. The local standard practices were applied in field management.

### Phenotypic evaluation and statistical analysis

At maturity, eight plants from each line of 13CM, as well as the parents, were randomly selected for phenotypic evaluation. Traits including plant height (PHT), productive tiller number (PTN), spike length (SL), fertile spikelet number (FSN), and GNS were measured manually. The average values of these traits from the eight selected plants in each line were utilized for statistical analysis. Additionally, after air-drying, grain length (GL), grain width (GW), the ratio of GL to GW (GL/GW), and thousand grain weight (TGW) were measured. The spike compactness (SC) was calculated by dividing FSN by SL. GNFS was calculated by dividing GNS by FSN. The detailed method was conducted as described previously (Ji et al., 2021).

Descriptive statistics, Pearson’s correlation analysis, normal distribution, and Student’s *t*-test were carried out using SPSS v24.0 (SPSS, Chicago, USA). The QTL IciMapping v4.2 software (https://isbreeding.caas.cn/rj/qtllcmapping/) was used to calculate the broad-sense heritability (*H* ^2^) and the best linear unbiased estimation (BLUE) dataset for each trait. OriginPro v2019 (https://www.originlab.com/) was employed to create the histogram distribution, scatter plot, and box plot. The Pearson’s correlation coefficients were utilized to examine the correlations between GNS and the other traits. Furthermore, by considering the genotypes of the flanking markers, lines harboring different alleles were compared using Student’s *t*-test, with a significance level set at *P* < 0.05.

### BSE-Seq analysis

The high-quality genomic DNA from 13CM lines and the parents was extracted by a modified hexadecyltrimethylammonium bromide (CTAB) method. Based on the phenotypic data obtained in E1–E6, lines in each environment were rearranged from low to high. To construct extreme mixing pools, 30 lines within each of two tails with stable phenotype in at least four of the six environments were selected. Two pools (GNS-H and GNS-L) were bulked using an equal amount (1 µg) of DNA from the selected 30 individuals. The two pools and the parents were utilized for BSE-Seq analysis performed by Bioacme Biotechnology Co., Ltd. (Wuhan, China).

The raw data from this study have been deposited in the Genome Sequence Archive (Chen et al., 2021) at the National Genomics Data Center (CNCB-NGDC Members and Partners, 2022), which is a part of the China National Center for Bioinformation/Beijing Institute of Genomics, Chinese Academy of Sciences (GSA: CRA008821 for ZKM13F10 and CM42, CAR009113 for GNS-H and GNS-L). These datasets are publicly accessible and can be found at https://ngdc.cncb.ac.cn/gsa. The processing of raw data was performed according to the previously method (Ji et al., 2023). In this study, two methods Euclidean distance (ED) and Δ(SNP-index) were employed to identify SNP and InDel sites between the paired pools. The detailed analytical method was described previously (Yu et al., 2022).

### Development of molecular markers

Based on the BSE-Seq data, SNP/InDel in the associated genomic regions between the parents and extreme pools were converted to develop Kompetitive Allele-specific PCR (KASP) markers for genetic map construction. Common primers were designed from Triticae Multi-omics Center (http://202.194.139.32/). FAM and HEX probe sequences were added to the 5’ end of primers. The KASP genotype identification was performed in the QuantStudio™ 3 Real-Time PCR system designed by Thermo Fisher Scientific, with a reaction mixture containing 5 µL 2× main mixture, 0.8 µL primer mixture, 3 µL ddH_2_O, and 2 µL DNA template (50 ng/mL–100 ng/mL). The conditions and procedures for touchdown PCR was referred to Yu et al. (2022).

### Genetic map construction and QTL detection

The genetic map was constructed by JoinMap v4.1, and the QTL was detected by QTL IciMapping v4.2 (Meng et al., 2015). Markers that co-localized with others and had a missing rate more than 20% were discarded. The maximum likelihood mapping algorithm and Kosambi’s mapping function were utilized to establish marker order and calculate marker distance. QTL detection in each environment was conducted using the QTL IciMapping v4.2 software based on the Inclusive Composite Interval Mapping (ICIM) method in the Biparental Population (BIP) module, and the LOD threshold was set as 2.5. The interaction of QTL×environment (QE) was performed using QTL IciMapping v4.2 according to Multi-Environment Trails module (LOD = 2.5, PIN = 0.001, and step = 4 cM). QTL repeatedly identified in at least three environments were treated as stable. Moreover, QTL explaining more than 10% of the phenotypic variation was considered as major. Confidence intervals were estimated by the position ± 1 LOD. QTL with overlapping confidence intervals were considered equivalent and named according to the international genetic naming rules (http://wheat.pw.usda.gov/ggpages/wgc/98/Intro.htm), where ‘cib’ represents ‘Chengdu Institute of Biology’.

### Haplotype analysis

Haplotypes at the crucial regions of the major QTL were analyzed based on the resequencing data of 145 landmark cultivars in China (http://wheat.cau.edu.cn/Wheat_SnpHub_Portal/). Subsequently, a natural population comprising 321 wheat accessions was used to conduct haplotype analysis. These accessions were planted in Shifang during the 2022–2023 growing season. The planting and phenotypic evaluation were conducted following the same protocols as the 13CM lines.

### Prediction of candidate genes

Based on the mapping results, the physical positions of the flanking markers were converted from IWGSC RefSeq v1.0 to v2.1 using the Triticae Multi-omics Center (Zhu et al., 2021). The annotation and function of the genes located between the flanking markers were analyzed using Uniport (https://www.uniprot.org/). The expression pattern of the candidate genes was obtained from expVIP (http://www.wheat-expression.com/). These expression data were normalized using the ZeroToOne method and further presented in the HeatMap drawn by TBtools (Chen et al., 2020). The orthologues from rice (*Oryza sativa* L. *Japonica* group) and *Arabidopsis thaliana* were identified using Ensembl Plants (https://plants.ensembl.org/Triticum_aestivum/Info/Index). The functional information of these orthologues was obtained from China Rice Data Center (https://www.ricedata.cn/) for rice orthologues and *tair* (https://www.arabidopsis.org/) for *Arabidopsis* orthologues. In addition, based on the BSE-Seq data, nonsynonymous SNPs present in the exon regions of genes within the target regions were collected.

## Results

### Phenotypic performance

The GNS of ZKM13F10 was higher than that of CM42 in most environments. Significant differences in GNS between ZKM13F10 and CM42 were observed in E1, E4, E7 and the BLUE dataset (*P* < 0.05 or *P* < 0.01) (**Table 1**). In the 13CM population, GNS showed extensive and significant variation. Based on the BLUE dataset, the range of GNS variation was 42.86–72.47. The estimated value of *H* ^2^ of GNS was 0.83, indicating that GNS was mainly controlled by genetic factors. The continuous distribution of GNS across eight environments and the BLUE dataset showed that it is a typical quantitative trait controlled by multiple genes (**Supplementary Figure 1** and **Table 1**). In multiple environments, the significant Pearson correlation of GNS ranged from 0.27 to 0.99 (*P* < 0.001) (**Supplementary Figure 1**).

**Table 1:** Phenotypic variation and heritability (*H* ^2^) of grain number per spike (GNS) of the parents and 13CM lines in eight environments and the BLUE dataset.

Env.	Parents		13CM lines					*H* ^2^
	ZKM13F10	CM42	Range	Mean ± SD	SK.	Ku.	CV (%)	
E1	77.67	49.67**	43.17–86.50	60.75 ± 6.21	0.40	0.92	10.2	0.83
E2	N	N	42.60–79.25	59.56 ± 5.88	0.15	0.14	9.6	
E3	68.00	65.33	42.30–81.09	60.85 ± 0.38	0.20	0.30	9.8	
E4	64.50	50.00*	43.45–80.84	59.46 ± 0.40	0.30	0.30	10.5	
E5	60.30	63.71	38.67–71.17	56.15 ± 0.37	−0.14	0.22	10.1	
E6	N	N	41.30–75.82	57.30 ± 0.41	−0.06	0.22	10.9	
E7	63.83	48.88**	39.32–76.16	56.84 ± 0.41	0.17	0.77	11.1	
E8	63.17	53.00	29.00–80.88	60.83 ± 0.47	−0.37	0.86	11.9	
BLUE	64.24	50.19*	42.86–72.47	58.52 ± 0.30	−0.03	0.27	7.9	

Env., environment; BLUE, best linear unbiased estimation; SD, standard deviation; SK., skewness; Ku., kurtosis; CV, coefficient of variation; N, the data were missed; H^2^, broad-sense heritability; * and ** represent significant at P < 0.05 and P < 0.01.

### Correlation analysis between GNS and other yield-related traits

**Figure 1:**
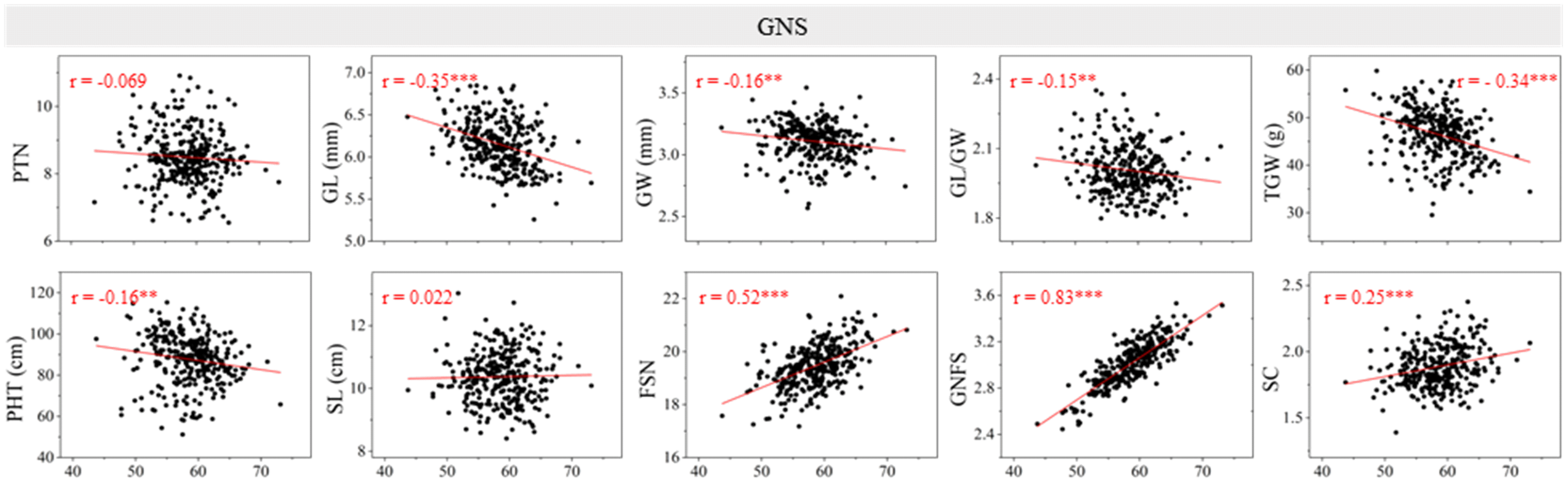
Coefficients of the pairwise Pearson’s correlations between grain number per spike (GNS) and other yield-related traits in the 13CM population. The traits include productive tiller number (PTN), grain length (GL), grain width (GW), GL/GW, thousand grain weight (TGW), plant height (PHT), spike length (SL), fertile spikelet number per spike (FSN), grain number per fertile spikelet (GNFS), and spike compactness (SC) (the coefficient of the pairwise Pearson’s correlations between GNS and FSN, GNFS have been published in Jiang et al., 2023). ** and *** represent significance at *P* < 0.01 and *P* < 0.001.

The Pearson’s correlation between GNS and other yield-related traits was evaluated using the BLUE dataset (**Figure 1**). Significant and negative correlations were detected between GNS and GL, GW, GL/GW, TGW, and PHT (*P* < 0.01 or *P* < 0.001). Moreover, GNS was significantly (*P* < 0.001) and positively correlated with FSN, GNFS, and SC. No significant correlation was observed between GNS and PTN (*r* = −0.069) or SL (*r* = 0.022). The correlation coefficient between GNS and GNFS was highest (*r* = 0.83).

### BSE-Seq analyses

Based on the BSE-Seq data from four libraries, genomic regions associated with GNS were detected (**Supplementary Table 1**). After filtering, the numbers of clean reads in the four libraries were 77,283,512 (ZKM13F10), 91,988,190 (CM42), 119,966,894 (GNS-H), and 171,166,254 (GNS-L), respectively. This result indicates that the data volume is sufficient for the subsequent analysis (**Supplementary Table 2**). Approximately 99.44% or higher of the captured reads were successfully mapped to the reference genome. The average sequencing depths ranged from 20.03× to 64.96×. Moreover, the coverage ≥5× varied from 60.38% to 78.15% in the four libraries, demonstrating high quality and adequate sequencing depth for BSE-Seq analysis. A total of 5,969,324 SNPs were identified in the dataset, and the number of SNPs per chromosome ranged from 58,929 to 630,245.

**Figure 2:**
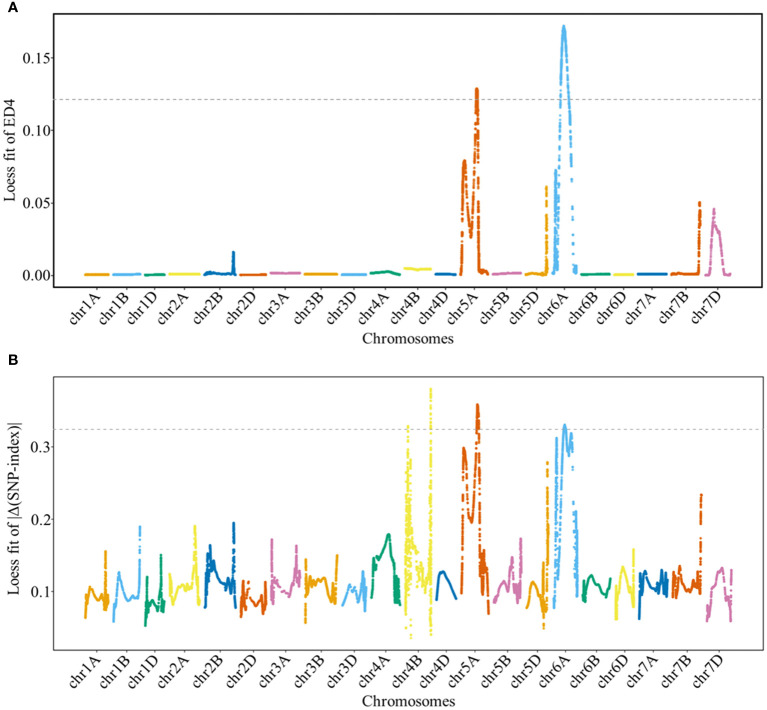
Locally weighted scatterplot smoothing (LOESS) fitting Manhattan plot for grain number per spike (GNS). Panels **(A, B)** show the LOESS fits of ED^4^ and |Δ(SNP-index)| for GNS, respectively. The cutoff values for the two methods are indicated by the dotted lines, with threshold values of 0.1214 and 0.3243 for the LOESS fits of ED^4^ and |Δ(SNP-index)|, respectively.

The ED and Δ(SNP-index) methods were used to detect genomic regions associated with GNS. Based on these results, genomic regions associated with GNS were detected on chromosomes 5A and 6A by ED and on chromosomes 4B, 5A, and 6A by Δ(SNP-index), respectively (**Figure 2** and **Supplementary Table 3**). Specifically, the overlapping physical intervals detected by both methods were found in the range of 404.14 Mb–440.88 Mb on chromosome 5A and in 265.97 Mb–320.49 Mb on chromosome 6A.

### Genetic map construction and QTL analysis

To confirm the preliminarily detected genomic regions associated with GNS, the polymorphic SNP sites within expanded regions (chr5A: 332.84 Mb–532.48 Mb; chr6A: 80.04 Mb–486.84 Mb) were converted into KASP markers (**Supplementary Tables 4**, **12**). The phenotypic data evaluated in eight environments and the combined analysis (the BLUE dataset was set as an additional environment) were used for QTL mapping.

**Figure 3:**
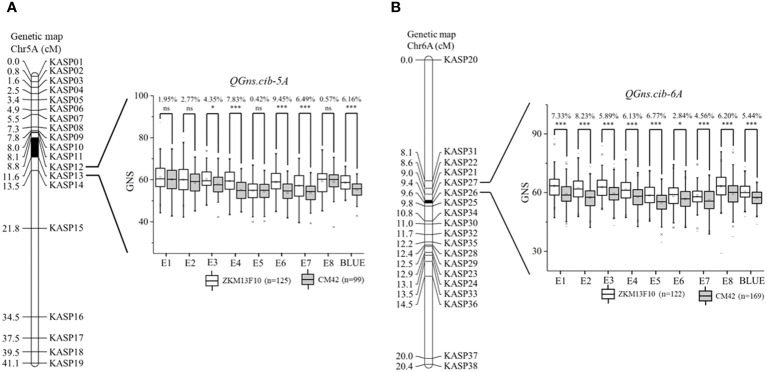
Genetic maps of the major quantitative trait loci (QTL) *QGns.cib-5A* **(A)** and *QGns.cib-6A* **(B)** and their effects on grain number per spike (GNS). The black area represents the genetic intervals of *QGns.cib-5A* and *QGns.cib-6A*. The effects of the major QTL on GNS are shown as box plots, calculated after grouping the 13CM population into two classes based on the KASP marker. ZKM13F10 and CM42 indicate the lines with and without positive alleles of *QGns.cib-5A* and *QGns.cib-6A*, respectively. ns, *, and *** represent significance at P > 0.05, P < 0.05, and P < 0.001.

**Figure 4:**
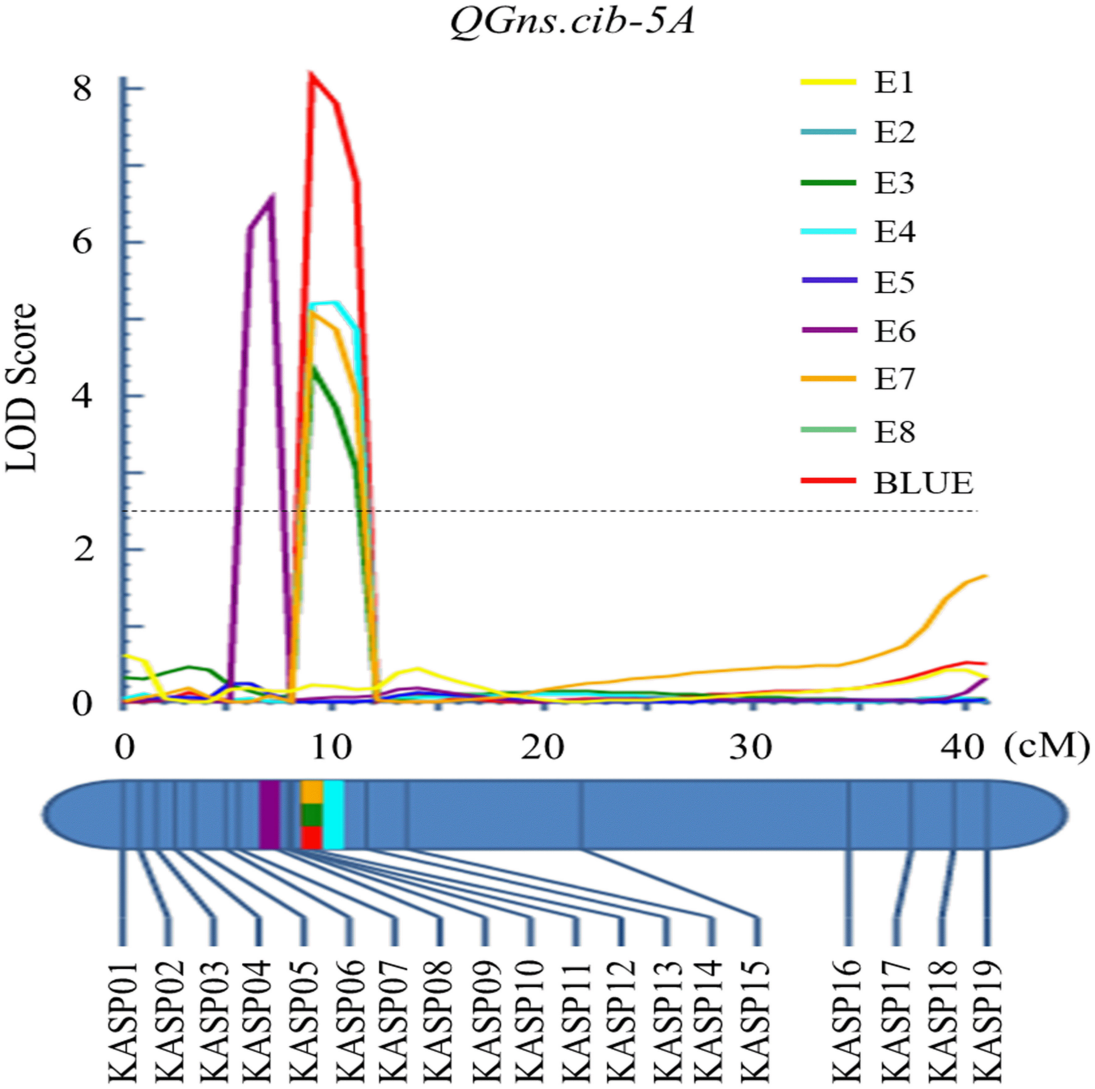
Logarithm of the odds (LOD) profile of QGns.cib-5A in the 13CM population using the inclusive composite interval mapping (ICIM) method.

For chromosome 5A, 19 KASP markers were successfully developed to construct a genetic map with a length of 41.1 cM. According to this map, a major and stable additive QTL *QGns.cib-5A* was detected in five environments including the BLUE dataset (**Figures 3A** and **4**). It explained 8.46%–14.43% of phenotypic variance, and the LOD values varied from 4.35 to 8.16. The favorable allele of *QGns.cib-5A* was contributed by ZKM13F10, and this QTL was located in a 2.8-cM genetic interval between the markers *KASP12* and *KASP13* (**Table 2**).

**Table 2:** Quantitative trait loci (QTL) on chromosomes 5A and 6A for grain number per spike (GNS) identified across multiple environments and the BLUE dataset in the 13CM population.

QTL	Env.	Genetic interval (cM)	Flanking markers	LOD	PVE (%)	Add	Physical position (Mb)
*QGns.cib-5A*	E3	8.50–10.50	*KASP12–KASP13*	4.35	8.46	1.70	435.62–441.15
	E4	8.50–10.50	*KASP12–KASP13*	5.20	9.58	2.00	
	E6	8.50–10.50	*KASP07–KASP08*	6.55	12.24	2.22	
	E7	5.50–7.50	*KASP12–KASP13*	5.07	9.29	1.96	
	BLUE	8.50–10.50	*KASP12–KASP13*	8.16	14.43	1.78	
*QGns.cib-6A*	E5	8.50–9.50	*KASP26–KASP27*	30.80	11.47	4.05	236.95–263.29
	E7	8.50–9.50	*KASP26–KASP27*	3.82	5.44	1.40	
	E8	8.50–9.50	*KASP26–KASP27*	22.95	5.58	4.36	
	BLUE	8.50–9.50	*KASP26–KASP27*	9.04	12.38	1.53	

Env., environment; PVE, phenotypic variation explained; LOD, logarithm of the odd; Add, additive effect (positive values indicate that the alleles from ZKM13F10 increases the trait scores, and negative values indicate that the allele from CM42 increases the trait scores); BLUE, best linear unbiased estimation.

**Figure 5:**
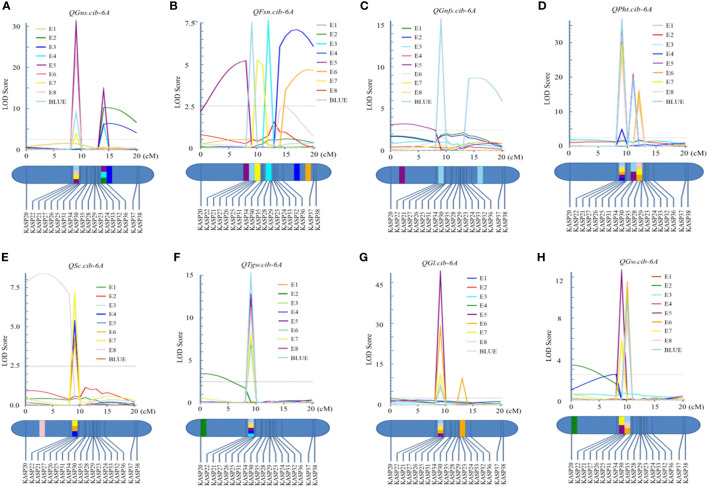
Logarithm of the odds (LOD) profile of *QGns.cib-6A* **(A)**, *QFsn.cib-6A* **(B)**, *QGnfs.cib-6A* **(C)**, *QPht.cib-6A* **(D)**, *QSc.cib-6A* **(E)**, *QTgw.cib-6A* **(F)**, *QGl.cib-6A* **(G)**, and *QGw.cib-6A* **(H)** in the 13CM population using the inclusive composite interval mapping (ICIM) method.

For chromosome 6A, 19 KASP markers were developed and the genetic map spanned 20.4 cM in length. *QGns.cib-6A*, a major and stable additive QTL, was identified in E5, E7, E8, and the BLUE dataset (**Figures 3B** and **5A**). It explained 5.44%–12.38% of phenotypic variance with the LOD values ranging from 3.82 to 30.80. The favorable allele of *QGns.cib-6A* was contributed by ZKM13F10. The QTL was located in a 0.2-cM genetic interval between the markers *KASP26* and *KASP27* (**Table 2**).

In addition, seven QTL were identified between the markers *KASP26* and *KASP27* on chromosome 6A (**Supplementary Table 5**). Three major and stable QTL (*QTgw.cib-6A*, *QGl.cib-6A*, and *QGw.cib-6A*) related to grain size and weight were detected (**Figures 5F–H**). *QTgw.cib-6A* was detected in five environments and the BLUE dataset and explained 10.26%–19.94% of phenotypic variance, with the LOD values ranging from 7.20 to 15.02. The *QGl.cib-6A* (LOD = 6.01–48.38; PVE = 8.68%–14.77%) was detected in four environments and the BLUE dataset. *QGw.cib-6A* (LOD = 5.70–12.48; PVE = 8.12%–16.74%) was detected in four environments and the BLUE dataset. The favorable alleles of *QTgw.cib-6A*, *QGl.cib-6A*, and *QGw.cib-6A* were all contributed by CM42.

For grain number-related traits, three QTL *QFsn.cib-6A*, *QGnfs.cib-6A*, and *QSc.cib-6A* were also identified (**Figures 5B, C, E**). *QFsn.cib-6A* (LOD = 5.22–7.40; PVE = 7.48%–10.34%), a major and stable QTL, was detected in E5, E7, and the BLUE dataset. *QSc.cib-6A* (LOD = 4.18–7.07; PVE = 6.21%–9.86%), a stable QTL, was identified in E4, E6, and E7. *QGnfs.cib-6A* (LOD = 3.17–15.55; PVE = 4.69%–4.99%), a minor QTL, was detected in E5 and the BLUE dataset. The favorable allele of *QFsn.cib-6A*, *QGnfs.cib-6A*, and *QSc.cib-6A* was all contributed by ZKM13F10.

Meanwhile, *QPht.cib-6A*, a stable QTL, was detected in six environments (**Figure 5D**). It explained 6.62%–8.39% of phenotypic variance with the LOD values ranging from 4.80 to 36.31. The favorable allele of *QPht.cib-6A* was contributed by CM42.

Based on the mapping result, eight QTL, *QGns.cib-6A*, *QTgw.cib-6A*, *QGl.cib-6A*, *QGw.cib-6A*, *QFsn.cib-6A*, *QGnfs.cib-6A*, *QSc.cib-6A*, and *QPht.cib-6A*, were detected in the same interval. Temporarily, we designated this common locus as *QClu.cib-6A.*

In the QE interaction analysis, a total of 19 QTL were detected, including the nine QTL identified in the single-environment analysis. Except for *QGns.cib-5A*, *QGw.cib-6A*, and *QPht.cib-6A*, the PVE (A) of the remaining six QTL were significantly smaller than that of PVE (AE), indicating that these QTL were not stable across environments (**Supplementary Table 6**). No epistatic QTL were found in this study (data not shown).

### Effects of major QTL on corresponding traits

By analyzing the genotyping results of the flanking markers *KASP12* and *KASP26*, the effects of *QGns.cib-5A* and *QGns.cib-6A* on GNS were examined. For *QGns.cib-5A*, lines with homozygous alleles from ZKM13F10 and CM42 were divided into two groups. Significant differences (*P* < 0.05 or *P* < 0.001) in GNS were observed between these groups. *QGns.cib-5A* was found to significantly increase GNS by 4.35%–9.45% across five environments (E3, E4, E6, E7, and the BLUE dataset) (**Figure 3A**). For *QGns.cib-6A*, significant differences (*P* < 0.001) in GNS were observed between the groups in all environments. *QGns.cib-6A* significantly increased GNS by 2.84%–8.23% (**Figure 3B**).

Effects of *QClu.cib-6A*, a QTL cluster, on other seven yield-related traits except GNS were assessed. For three grain size and weight traits, significant differences (*P* < 0.01 or *P* < 0.001) between groups in all or eight environments were detected and *QClu.cib-6A* significantly increased TGW, GL, and GW by 4.66%–15.92%, 1.55%–4.66%, and 1.70%–6.13%, respectively (**Supplementary Figures 2E-G**). For three grain number-related traits, significant differences (*P* < 0.05, *P* < 0.01, or *P* < 0.001) between groups were detected in seven or six environments and *QClu.cib-6A* significantly increased FSN, GNFS, and SC by 2.72%–3.44%, 2.34%–3.76%, and 4.90%–7.12%, respectively (**Supplementary Figures 2B–D**). Meanwhile, significant differences (*P* < 0.05 or *P* < 0.001) on plant height were found between groups in all environments, and *QClu.cib-6A* significantly increased PHT by 3.82%–11.61% (**Supplementary Figure 2A**).

### Effects of *QGns.cib-5A* and *QGns.cib-6A* on other yield-related traits

To detect the effects of *QGns.cib-5A* and *QGns.cib-6A* on other yield-related traits, the 13CM lines were divided into two groups based on the marker’s spectra of *KASP12* and *KASP26*, respectively. For *QGns.cib-5A*, the comparative analysis between the two groups based on the BLUE dataset showed that *QGns.cib-5A* had significant effects on PTN, FSN, SL, and GL/GW (*P* < 0.05, *P* < 0.01, or *P* < 0.001) (**Supplementary Figure 3**). Significant differences in PTN and SL were observed between the two groups for *QGns.cib-6A* (*P* < 0.001) (**Supplementary Figure 4**).

### Additive effect of *QGns.cib-5A* and *QGns.cib-6A*

**Figure 6:**
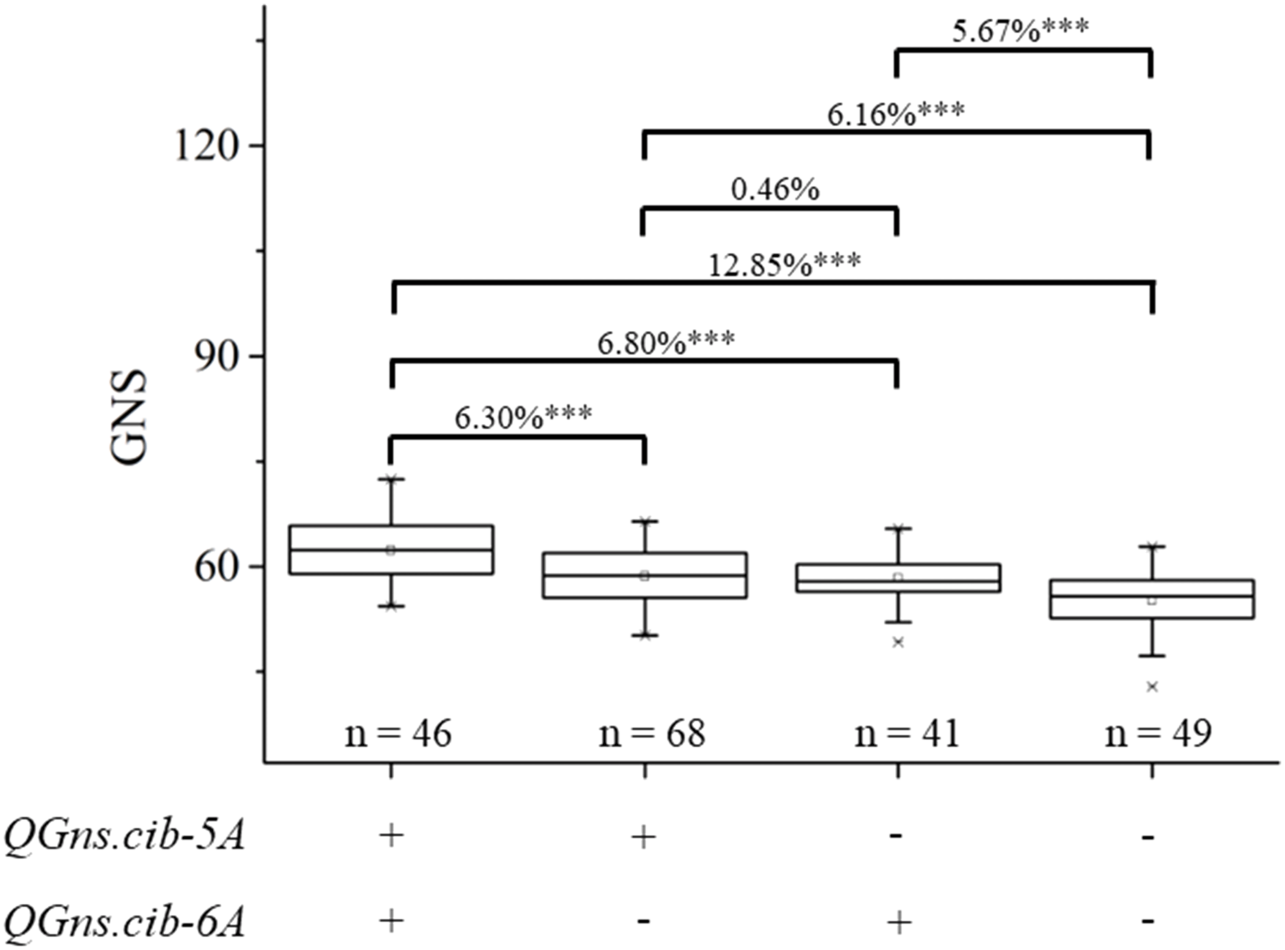
Additive effects of *QGns.cib-5A* and *QGns.cib-6A* on grain number per spike (GNS) in the 13CM population. “+” and “–” represent lines with the alleles from ZKM13F10 and CM42 of the target loci, respectively. *** represents significance at P < 0.001.

In the present study, two QTL *QGns.cib-5A* and *QGns.cib-6A* for GNS were identified. Subsequently, the additive effects of *QGns.cib-5A* and *QGns.cib-6A* on GNS in the 13CM population were analyzed. Compared with lines with unfavorable alleles, lines with a favorable allele of GNS at *QGns.cib-5A* or *QGns.cib-6A* significantly increased GNS by 6.16% (*P* < 0.001) or 5.67% (*P* < 0.001), respectively. Compared with lines carrying unfavorable alleles, lines with both favorable alleles exhibited a significant increase in GNS by 12.85% (*P* < 0.001) (**Figure 6**).

### Validation of *QGns.cib-5A* and *QGns.cib-6A* in different genetic backgrounds

**Figure 7:**
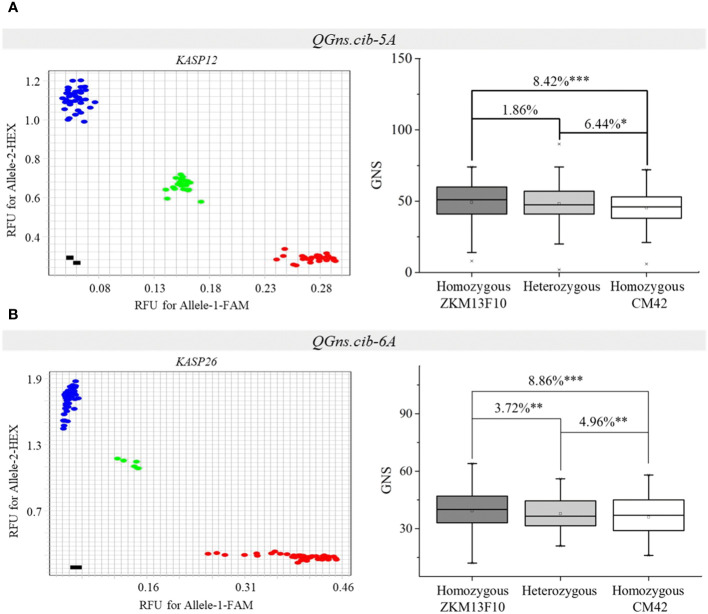
Validation of *QGns.cib-5A* **(A)** and *QGns.cib-6A* **(B)** in different genetic backgrounds. The fluorescence PCR genotyping results of the Kompetitive Allele-Specific PCR (KASP) markers *KASP12* and *KASP26* in the CZ5782 and CS352 populations, respectively. The effects of *QGns.cib-5A* and *QGns.cib-6A* on grain number per spike (GNS) in the CZ5782 and CS352 populations, respectively. *, **, and *** represent significance at *P* < 0.05, *P* < 0.01, and *P* < 0.001, respectively.

Two populations (CZ5782 and CS104) were used to evaluate the effects of *QGns.cib-5A* and *QGns.cib-6A* in different genetic backgrounds, respectively. *KASP12* (closely linked to *QGns.cib-5A*) and *KASP26* (tightly linked to *QGns.cib-6A*) were used for genotyping. For *KASP12*, polymorphism was detected in *QGns.cib-5A* between CM104 and ZM5782. For *KASP26*, polymorphism was detected in *QGns.cib-6A* between CM104 and SH352. According to the genotyping results, the F_2_ individuals from CZ5782 and CS104 were divided into three groups: individuals with a CM42 homozygous allele, individuals with a non-CM42 homozygous allele, and individuals with heterozygous allele. Significant differences (*P* < 0.05, *P* < 0.01, or *P* < 0.001) in GNS were identified between the groups with different alleles in both populations. Lines with the favorable and homozygous alleles significantly increased GNS by 6.44%–8.42% and 3.72%–8.86% in CZ5782 and CS104 populations, respectively (**Figure 7**).

### Candidate gene analysis of *QGns.cib-5A* and *QGns.cib-6A*

After screening the physical interval of *QGns.cib-5A* (435.62 Mb–441.15 Mb) using IWGSC RefSeq v2.1, 150 prediction genes including 76 high-confidence prediction genes were obtained (**Supplementary Table 7**). Spatial expression patterns showed that 26 genes were highly expressed in spike, indicating that they might participate in spike development (**Supplementary Figure 5**). In addition, according to gene annotation, and homologous gene function in rice and/or *Arabidopsis thaliana*, *TraesCS5A03G0562600* might be related to spike development. According to the BSE-Seq data, two SNPs and an InDel were identified between the two parents of *TraesCS5A03G0562600* (**Supplementary Table 8**).

For *QGns.cib-6A*, 144 prediction genes (including 35 high-confidence genes) were detected in the physical interval of 236.95 Mb–263.29 Mb using IWGSC RefSeq v2.1 (**Supplementary Table 7**). Expression patterns suggested that 16 genes were highly expressed in spike, indicating that they might be related to spike development (**Supplementary Figure 6**). Furthermore, according to the gene annotation and the homologous gene function in rice and/or *Arabidopsis thaliana*, *TraesCS6A03G0487300* and *TraesCS6A03G0492700* might participate in spike development. Based on the BSE-Seq data, an InDel and one InDel were found in the upstream and exon of the *TraesCS6A03G0487300* and *TraesCS6A03G0492700*, respectively.

### Haplotype analysis of *QGns.cib-5A* and *QGns.cib-6A*

**Figure 8:**
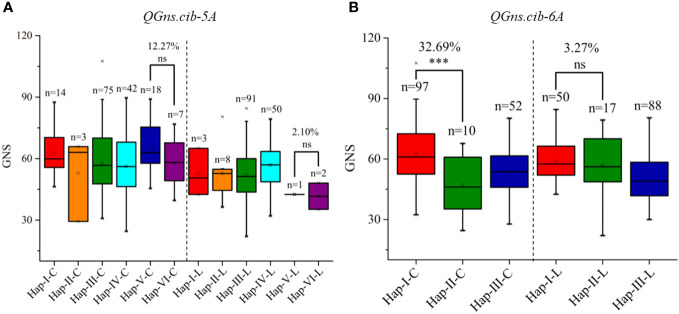
Haplotypes and their distribution frequency of *QGns.cib-5A* **(A)** and *QGns.cib-6A* **(B)** in 321 wheat accessions. ‘C’ and ‘L’ represent ‘cultivars’ and ‘landraces’, respectively; ns and *** represent significance at *P* > 0.05 and *P* < 0.001.

According to the high-quality resequencing data of 145 Chinese wheat accessions, the haplotypes in the key regions of *QGns.cib-5A* and *QGns.cib-6A* were analyzed. Six and three haplotypes were found in *QGns.cib-5A* and *QGns.cib-6A*, respectively (**Supplementary Figures 7**, **8**). For *QGns.cib-5A*, six KASP markers were successfully developed to differentiate the six haplotypes and used to perform the haplotype analysis in our natural population (321 wheat accessions). As expected, all six haplotypes were detected, namely, haplotype-I, -II, -III, -IV, -V, and -VI (**Supplementary Figure 9A** and **Supplementary Table 11**). Based on the association analysis result, GNS of accessions with hap-V (including ZKM13F10) was 12.27% and 2.10% higher than that of accessions with hap-VI (including CM42) in ‘Cultivars’ and ‘Landraces’, respectively (**Figure 8A**).

For *QGns.cib-6A*, three KASP markers were developed to distinguish the three haplotypes and haplotype analysis was carried out in 321 wheat accessions. As expected, three haplotypes (hap-I, -II, and -III) were detected (**Supplementary Figure S9B**; **Supplementary Table 11**). According to association analysis, significant difference on GNS was detected between accessions with hap-I (including ZKM13F10) and hap-II (including CM42) in ‘Cultivars’. However, a higher but non-significant difference was detected between the two haplotypes in ‘Landraces’ (**Figure 8B**).

## Discussion

### Comparison of the detected QTL to those reported in previous studies

In this study, two major and stable QTL, *QGns.cib-5A* and *QGns.cib-6A*, were identified on chromosomes 5A and 6A, respectively, using the BSE-Seq method and linkage analysis. To detect if they overlap with QTL reported in previous studies, we compared their physical intervals based on the CS reference genome (**Table 2** and **Supplementary Table 9**).

For *QGns.cib-5A*, 14 QTL controlling GNS on chromosome 5A were screened in previous studies. Among them, *QGN.perg-5A* (462.01 Mb) and an unnamed QTL (tightly linked marker *Xgwm186*, 472.16 Mb) are located near the physical interval of *QGns.cib-5A* (435.62 Mb–441.15 Mb) (Liu et al., 2006; Pretini et al., 2021). *QGns.cau-5A.2* (439.67 Mb) and an un-named QTL (*Xgwm415*–*Xgwm304*, 107.10 Mb–664.99 Mb) were overlapped with *QGns.cib-5A* (Su et al., 2009; Guan et al., 2018). However, the unnamed QTL (*Xgwm415*–*Xgwm304*, 107.10 Mb–664.99 Mb) was identified in a large interval and only detected in two environments. Another unnamed QTL (tightly linked marker *Xgwm186*, 472.16 Mb) was detected in only one environment, suggesting it was unstable. *QGns.cau-5A.2* was detected in six environments and located within the physical interval of *QGns.cib-5A*, but its PVE value is less than 10%, indicating that it is a minor QTL. *QGN.perg-5A* is located near *QGns.cib-5A* and was detected in three environments with a PVE value ranging from 14.6% to 17.5%, suggesting that it is a major and stable QTL. As a result, whether *QGns.cib-5A* is a novel QTL or allelic to the reported loci remains to be revealed.

For *QGns.cib-6A*, several cloned genes and QTL around the candidate region associated with GNS have been reported in previous studies (**Supplementary Tables 9**, **10**). *TaBT1-6A*, located near *QGns.cib-6A* and associated with grain size, weight, and grain total starch content, was identified (Wang et al., 2019). However, our remapping result showed that *TaBT1-6A* was not linked to *QGns.cib-6A*, suggesting that *TaBT1-6A* is not the candidate gene of *QGns.cib-6A*. Another gene, *TaGW2-6A*, is located within the physical interval of *QGns.cib-6A* and plays pleotropic effects on wheat agronomic traits (Su et al., 2011; Jaiswal et al., 2015). Based on an SNP site (−593 bp, A/G) in the promoter region, a KASP marker *TaGW2-6A-593* was employed (Su et al., 2011) (**Supplementary Table 12**). The remapping results indicated that *TaGW2-6A* was not linked to *QGns.cib-6A*, suggesting that *TaGW2-6A* is not the candidate gene of *QGns.cib-6A*. Meanwhile, no previously reported QTL for GNS overlapped with *QGns.cib-6A* (**Supplementary Table 9**), indicating that it may be a novel QTL.

### Relationships between GNS and TGW and pleiotropic effects of *QGns.cib-5A* and *QGns.cib-6A*

Generally, a tradeoff between grain number and grain weight is usually detected, which has been a major limitation in further breeding program. In the present study, the significant and negative correlations between GNS and TGW, GL, GW, and GL/GW supports the tradeoff effect. According to statistics, approximately 90% of the identified QTL controlling GNS have a negative and pleiotropic effect on TGW (Yang et al., 2021). As a result, QTL controlling GNS with no effect on TGW is essential for breeding. In this study, *QGns.cib-6A* showed a significant and negative effect on TGW, indicating a typical tradeoff effect between GNS and TGW (**Supplementary Figure 2E**). On the other hand, *QGns.cib-5A* had no significant effect on TGW (**Supplementary Figure 3G**). This suggests that *QGns.cib-5A* can increase GNS without reducing TGW and can be utilized in breeding program.

### *QGns.cib-5A* and *QGns.cib-6A* are the artificial selection loci during wheat improvement

During the long history of wheat domestication and selection, favorable haplotypes have been retained and enriched. However, the limited availability of genomic information has restricted access to haplotype information in wheat. Recently, more resequencing data of wheat materials have become available. For instance, the Wheat SnpHub Portal database has collected 13 resequencing datasets, encompassing 3,253 wheat accessions. This provides us the opportunity to analyze the haplotypes of a specific genomic region.

In the present study, the haplotypes for the crucial regions of *QGns.cib-5A* and *QGns.cib-6A* were analyzed using the resequencing data from 145 Chinese landmark cultivars (Hao et al., 2020). Based on the haplotype analysis of 321 wheat accessions, only one (0.65%) and two (1.29%) wheat accessions, respectively, were detected in hap-V (containing ZKM13F10) and hap-VI (containing CM42) in landraces, which suggests they were both rare haplotypes of *QGns.cib-5A*. In cultivars, the distribution frequency of the two haplotypes increased, with 18 accessions (hap-V, 11.32%) and 7 accessions (hap-VI, 4.40%), respectively. This finding indicates that both haplotypes have been artificially selected and enriched during wheat improvement. For *QGns.cib-6A*, 50 (32.26%) and 17 (10.97%) wheat accessions were found in hap-I (containing ZKM13F10) and hap-II (containing CM42) in landrace, respectively. Moreover, in cultivar, the distribution frequency of hap-I was doubled (97, 61.01%) whereas hap-II was retained (10, 6.29%). These results suggest that hap-I was enriched. Overall, both *QGns.cib-5A* and *QGns.cib-6A* appear to have been the targets of artificial selection in wheat improvement.

### Potential candidate genes for *QGns.cib-5A* and *QGns.cib-6A*

Within the physical interval of *QGns.cib-5A* and *QGns.cib-6A*, 76 and 35 high-confidence prediction genes were detected in the CS reference genome, respectively (**Supplementary Table 7**). Through spatiotemporal expression patterns, homology analysis, function annotation, and sequence difference analysis, we predicted *TraesCS5A03G0562600* as a potential candidate gene for *QGns.cib-5A*. *TraesCS5A03G0562600* is the orthologous gene of *AUXIN RESISTANT 4* (*AXR4*) in *Arabidopsis*, and it encodes the pseudomolecule protein AUXIN RESPONSE 4 (**Supplementary Table 7**). In *Arabidopsis*, *AXR4* participates in biological processes of auxin polar transport (Hobbie, 2006). Auxin plays a crucial role in regulating plant growth, including the development of reproductive organs (Lampugnani et al., 2013). The BSE-Seq data revealed that two SNPs exist in the exon region of *TraesCS5A03G0562600*, which may result in functional change of this gene.

For *QGns.cib-6A*, we predicted *TraesCS6A03G0487300* and *TraesCS6A03G0492700* as potential candidate genes. *TraesCS6A03G0487300* is the orthologous gene of *SPATULA* (*SPT*) in *Arabidopsis*, encoding the pseudomolecule protein basic helix–loop–helix (bHLH) DNA-binding superfamily (**Supplementary Table 7**). In *Arabidopsis*, *SPT* participates in biological processes of flower development, suggesting it involves in regulating seed number (Pfannebecker et al., 2017). The BSE-Seq data revealed the presence of one SNP in the upstream region and two SNPs in the downstream regions of *TraesCS6A03G0487300*, potentially resulting in changes in expression levels (**Table S8**). *TraesCS6A03G0492700* is the orthologous gene of *OsUBP15* in rice and *UBIQUITIN-SPECIFIC PROTEASE 15* (*UBP15*) in *Arabidopsis*, and it encodes the pseudomolecule protein ubiquitin carboxyl-terminal hydrolase. *OsUBP15* involves in regulating the number of lateral cells in the glume and TGW in rice (Shi et al., 2019). *UBP15* participates in biological processes of cell division, flower development, fruit development, leaf development, and protein de-ubiquitination in *Arabidopsis* (Wu et al., 2022). Meanwhile, according to BSE-Seq data, two InDels were detected between the parents of *TraesCS6A03G0492700* (**Supplementary Table 8**). In summary, *TraesCS5A03G0562600* may be the candidate gene for *QGns.cib-5A*, whereas *TraesCS6A03G0487300* and *TraesCS6A03G0492700* may be the candidate genes for *QGns.cib-6A*, and their further investigation through map-based cloning would be valuable.

## Data availability statement

The datasets presented in this study can be found in online repositories. The names of the repository/repositories and accession number(s) can be found in the article/**Supplementary Material**.

## Author contributions

CJ: Data curation, Formal Analysis, Investigation, Software, Validation, Writing – original draft. ZX: Data curation, Resources, Writing – review & editing. XF: Methodology, Software, Writing – review & editing. QZ: Data curation, Resources, Writing – review & editing. GJ: Data curation, Resources, Writing – review & editing. SL: Data curation, Software, Writing – review & editing. YW: Software, Visualization, Writing – review & editing. FM: Software, Writing – review & editing. YZ: Project administration, Supervision, Writing – review & editing. TW: Funding acquisition, Project administration, Writing – review & editing. BF: Funding acquisition, Project administration, Supervision, Writing – review & editing.
