# Supplementary material for: Genetic dissection of major QTL for grain number per spike on chromosomes 5A and 6A in bread wheat (Triticum aestivum L.)
Source: Front Plant Sci. 2024 Jan 8;14:1305547. doi: 10.3389/fpls.2023.1305547 (PMC10800429; doi:10.3389/fpls.2023.1305547)

Fig. S9 Haplotypes of *QGns.cib-5A* (a) and *QGns.cib-6A* (b) in 60 modern Chinese cultivars and a wheat mini-core collection containing 261 wheat materials. Yellow and blue indicate alleles that are identical to or different from those in IWGSC RefSeq v1.0 reference genome, respectively. Heterozygous sites are shown in red

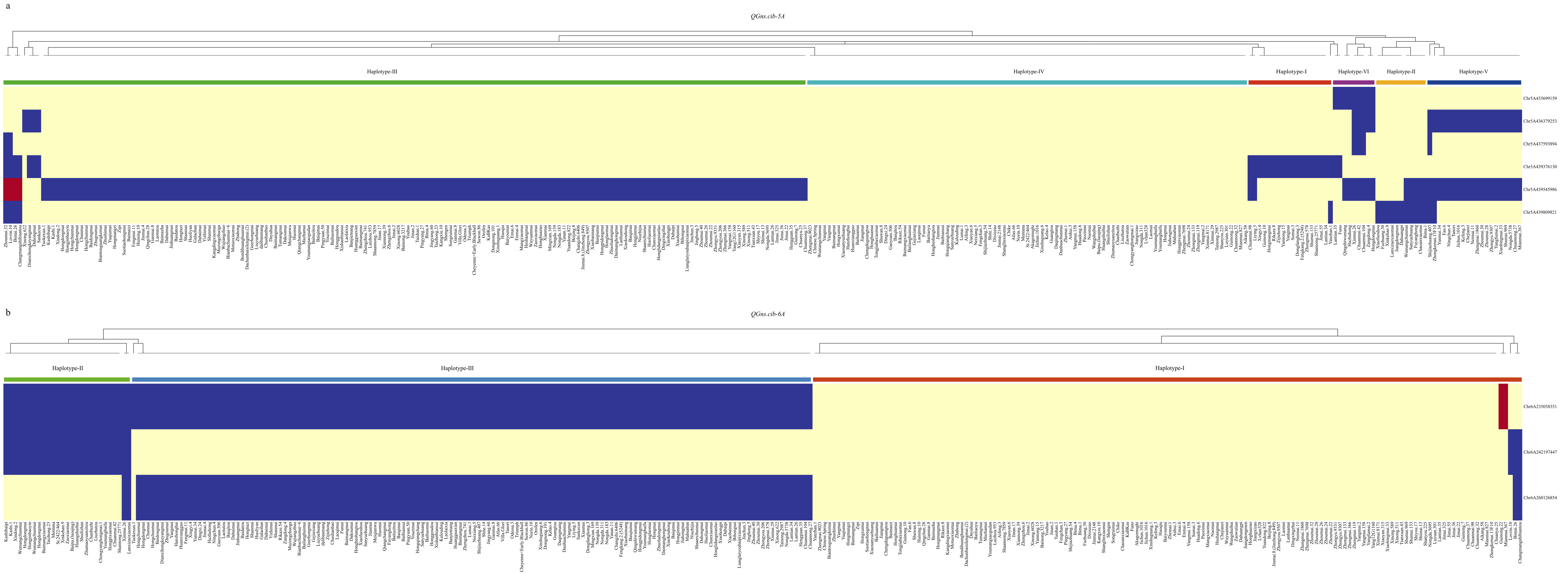

Supplement: Supplementary file 1 [file Image_1.pdf]
