# Supplementary material for: Genetic dissection of major QTL for grain number per spike on chromosomes 5A and 6A in bread wheat (Triticum aestivum L.)
Source: Front Plant Sci. 2024 Jan 8;14:1305547. doi: 10.3389/fpls.2023.1305547 (PMC10800429; doi:10.3389/fpls.2023.1305547)

**Fig. S7** Haplotypes of *Gns.cib-5A* in 145 landmark cultivars. Gray and blue indicate alleles that are identical to or different from those in the IWGSC RefSeq v1.0 reference genome, respectively. Heterozygous sites are shown in light blue, and deletions are shown in white.

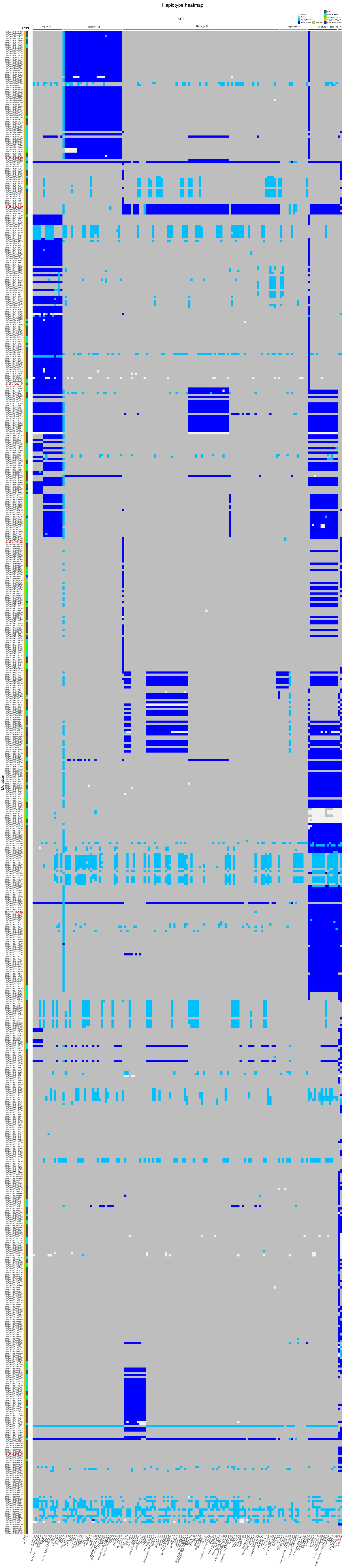

Supplement: Supplementary file 2 [file Image_2.pdf]
