# Supplementary material for: Genetic dissection of major QTL for grain number per spike on chromosomes 5A and 6A in bread wheat (Triticum aestivum L.)
Source: Front Plant Sci. 2024 Jan 8;14:1305547. doi: 10.3389/fpls.2023.1305547 (PMC10800429; doi:10.3389/fpls.2023.1305547)
Supplement: Supplementary file 4 [file Presentation_1.pptx]

## Slide 1
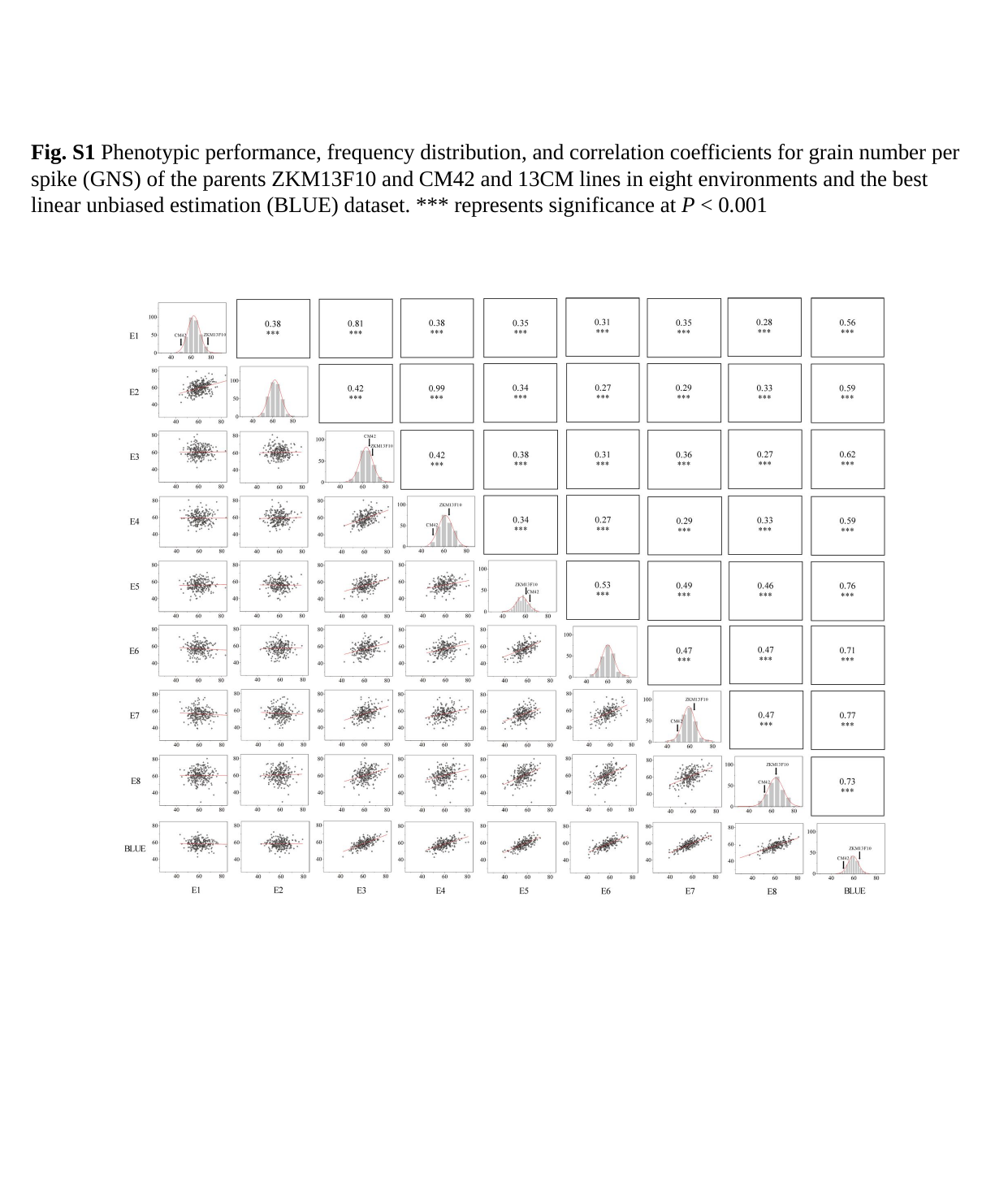

Fig. S1 Phenotypic performance, frequency distribution, and correlation coefficients for grain number per spike (GNS) of the parents ZKM13F10 and CM42 and 13CM lines in eight environments and the best linear unbiased estimation (BLUE) dataset. *** represents significance at P < 0.001

## Slide 2
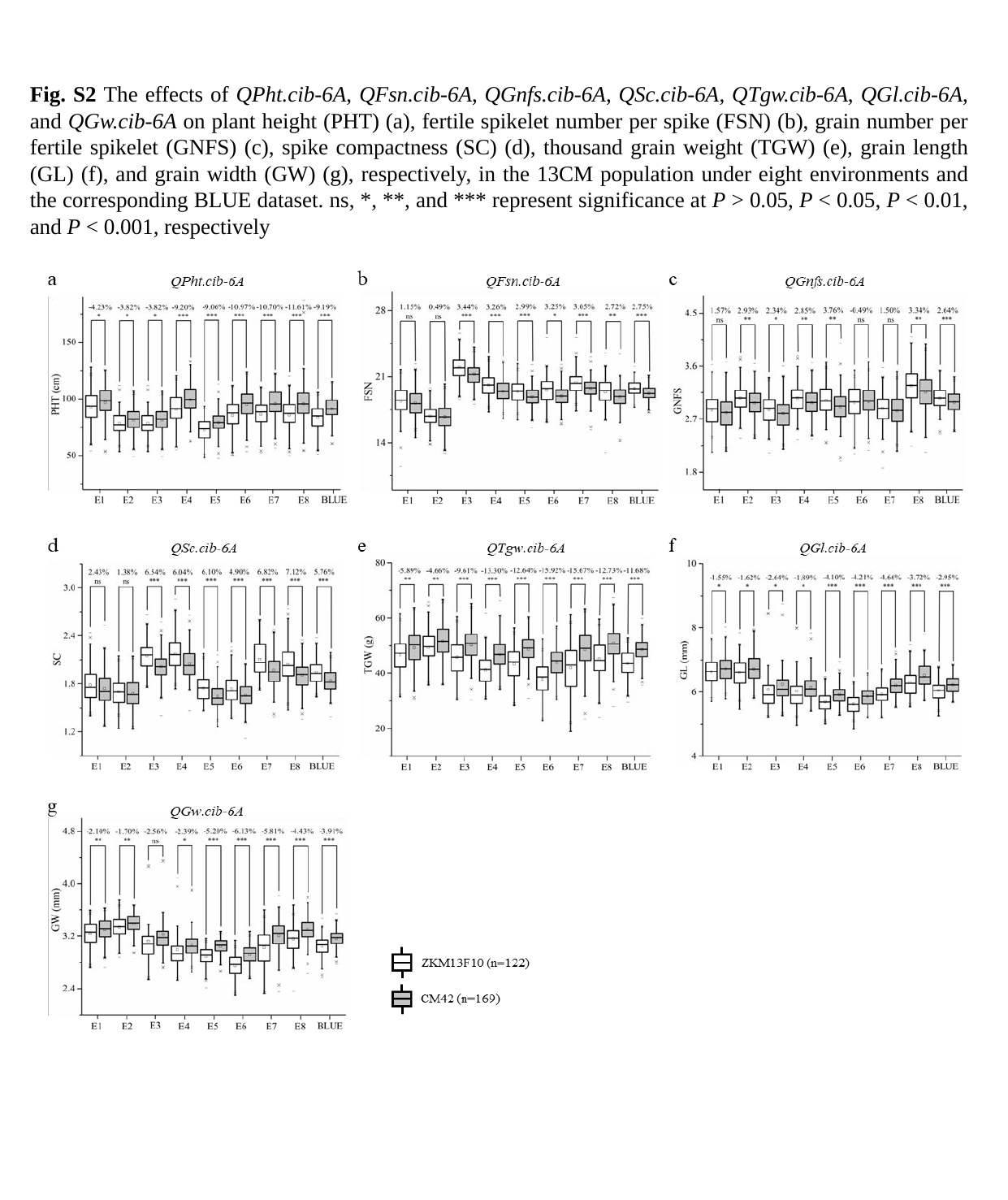

Fig. S2 The effects of QPht.cib-6A, QFsn.cib-6A, QGnfs.cib-6A, QSc.cib-6A, QTgw.cib-6A, QGl.cib-6A, and QGw.cib-6A on plant height (PHT) (a), fertile spikelet number per spike (FSN) (b), grain number per fertile spikelet (GNFS) (c), spike compactness (SC) (d), thousand grain weight (TGW) (e), grain length (GL) (f), and grain width (GW) (g), respectively, in the 13CM population under eight environments and the corresponding BLUE dataset. ns, *, **, and *** represent significance at P > 0.05, P < 0.05, P < 0.01, and P < 0.001, respectively

## Slide 3
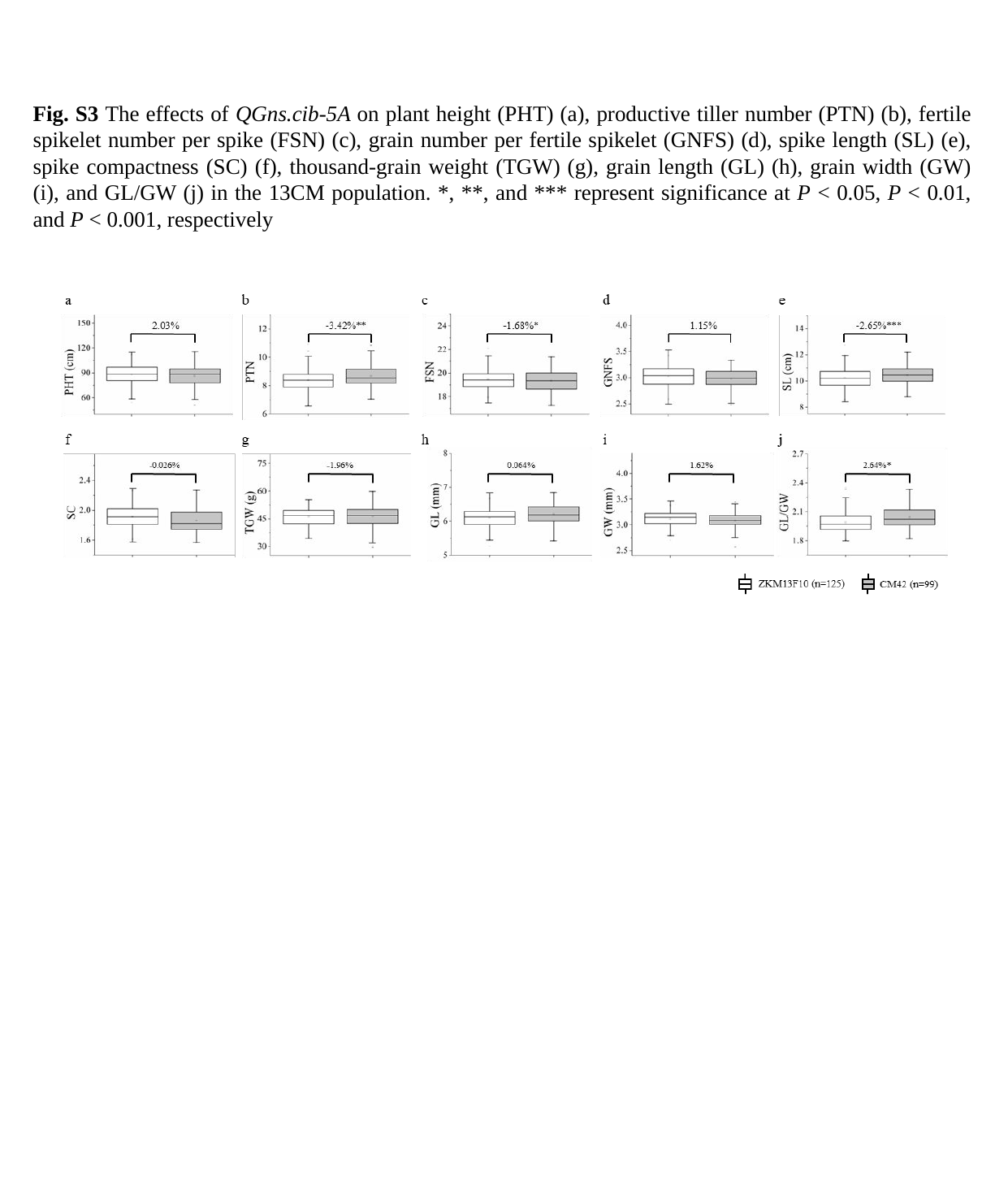

Fig. S3 The effects of QGns.cib-5A on plant height (PHT) (a), productive tiller number (PTN) (b), fertile spikelet number per spike (FSN) (c), grain number per fertile spikelet (GNFS) (d), spike length (SL) (e), spike compactness (SC) (f), thousand-grain weight (TGW) (g), grain length (GL) (h), grain width (GW) (i), and GL/GW (j) in the 13CM population. *, **, and *** represent significance at P < 0.05, P < 0.01, and P < 0.001, respectively

## Slide 4
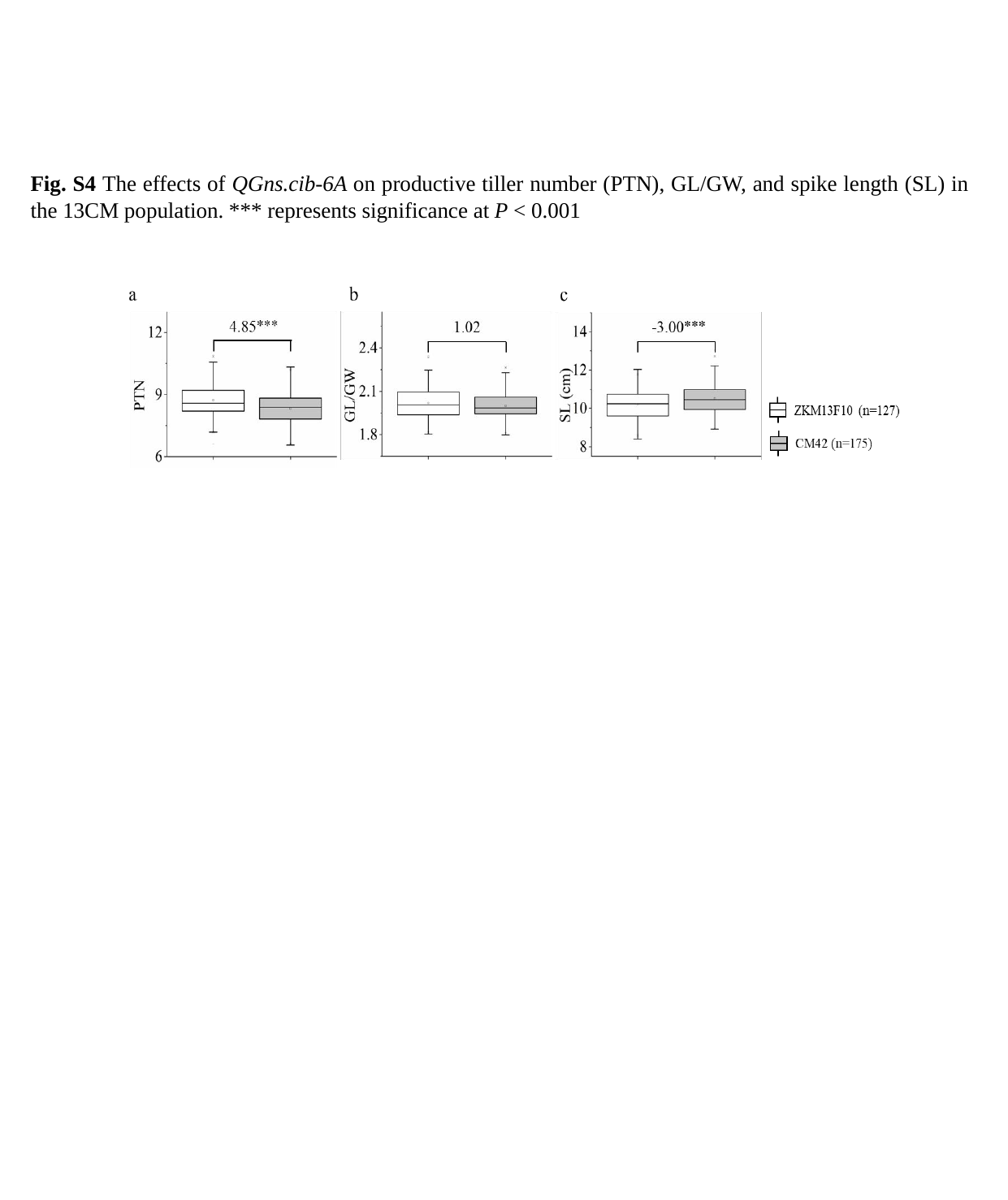

Fig. S4 The effects of QGns.cib-6A on productive tiller number (PTN), GL/GW, and spike length (SL) in the 13CM population. *** represents significance at P < 0.001

## Slide 5
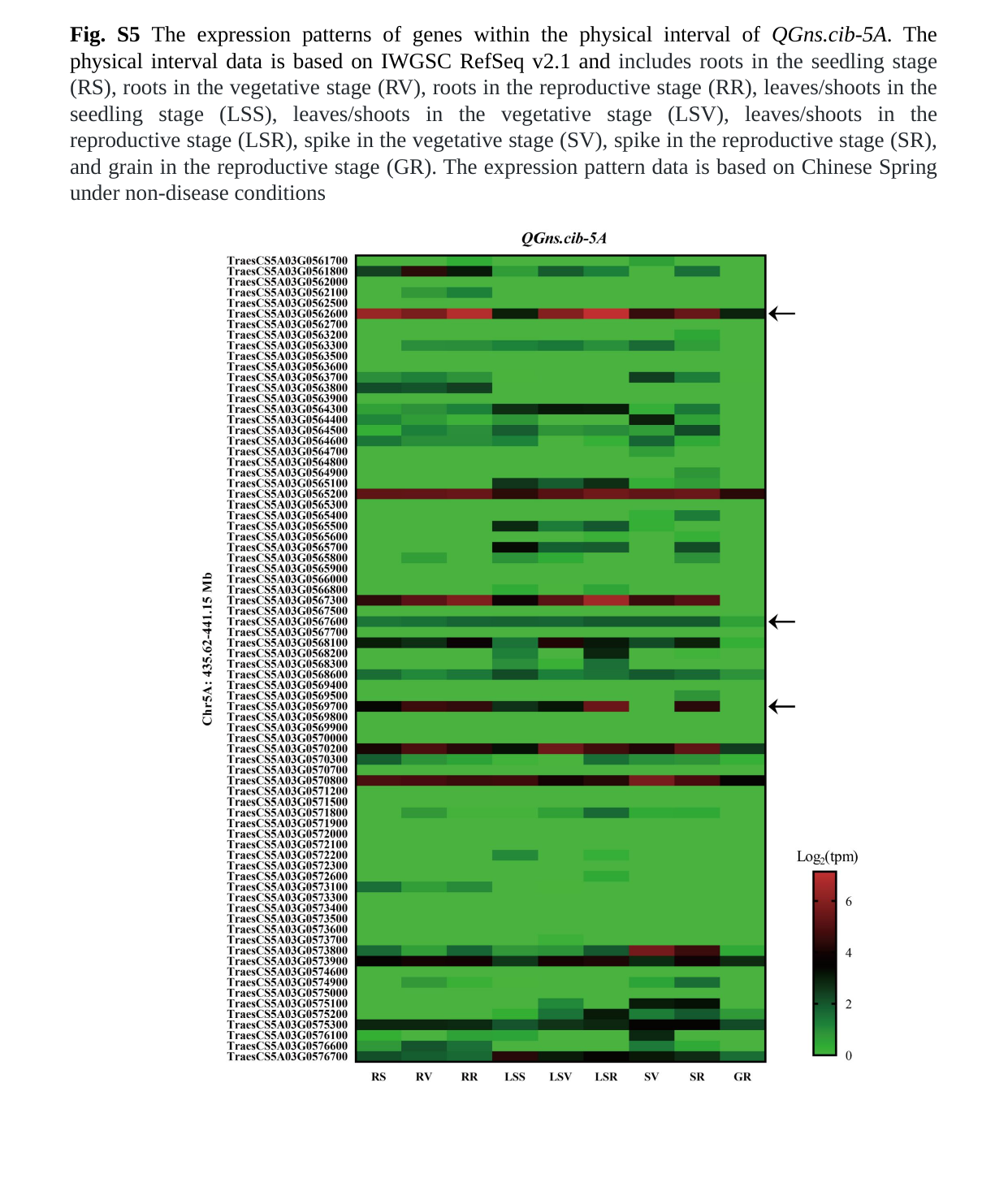

Fig. S5 The expression patterns of genes within the physical interval of QGns.cib-5A. The physical interval data is based on IWGSC RefSeq v2.1 and includes roots in the seedling stage (RS), roots in the vegetative stage (RV), roots in the reproductive stage (RR), leaves/shoots in the seedling stage (LSS), leaves/shoots in the vegetative stage (LSV), leaves/shoots in the reproductive stage (LSR), spike in the vegetative stage (SV), spike in the reproductive stage (SR), and grain in the reproductive stage (GR). The expression pattern data is based on Chinese Spring under non-disease conditions

## Slide 6
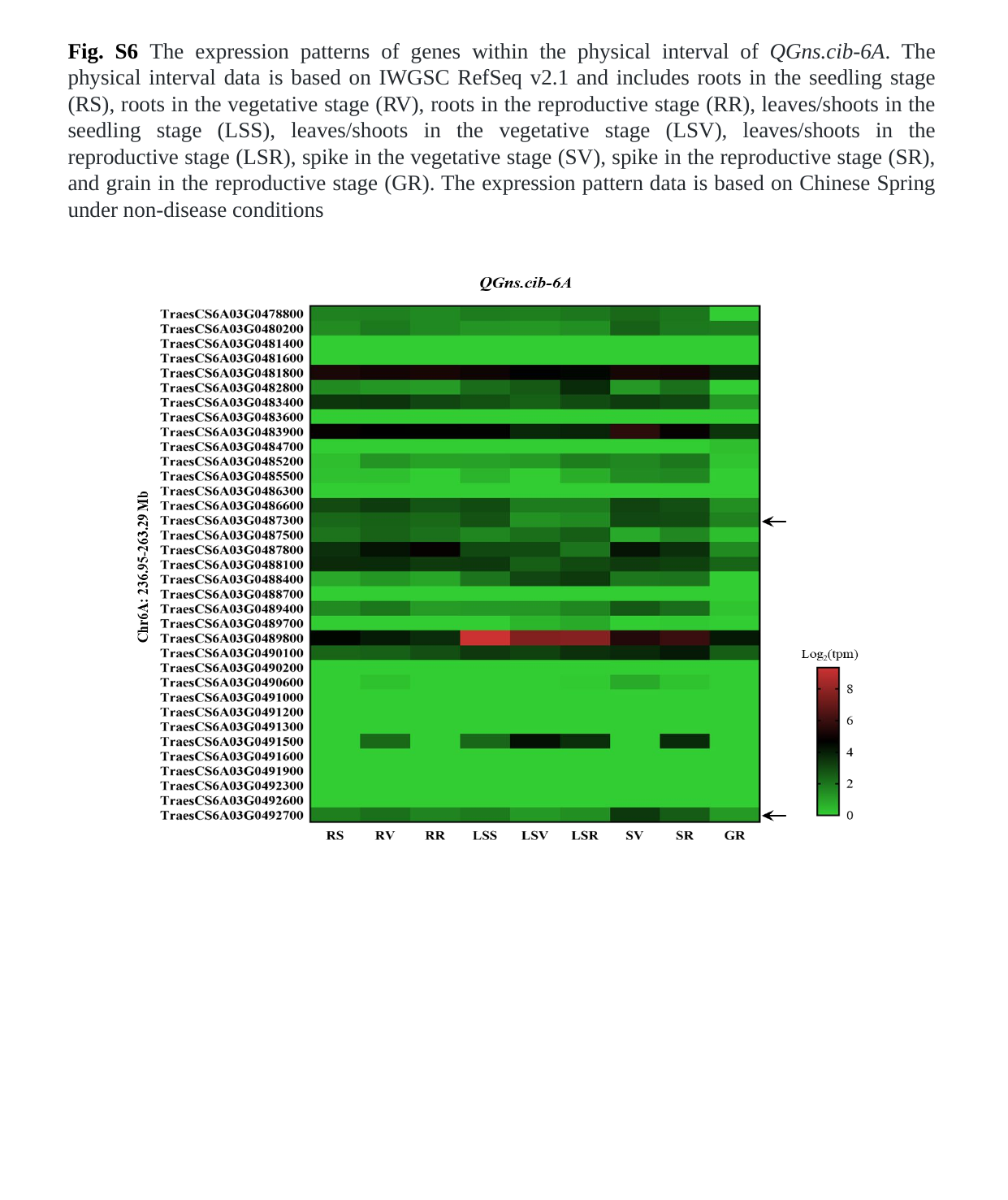

Fig. S6 The expression patterns of genes within the physical interval of QGns.cib-6A. The physical interval data is based on IWGSC RefSeq v2.1 and includes roots in the seedling stage (RS), roots in the vegetative stage (RV), roots in the reproductive stage (RR), leaves/shoots in the seedling stage (LSS), leaves/shoots in the vegetative stage (LSV), leaves/shoots in the reproductive stage (LSR), spike in the vegetative stage (SV), spike in the reproductive stage (SR), and grain in the reproductive stage (GR). The expression pattern data is based on Chinese Spring under non-disease conditions

## Slide 7
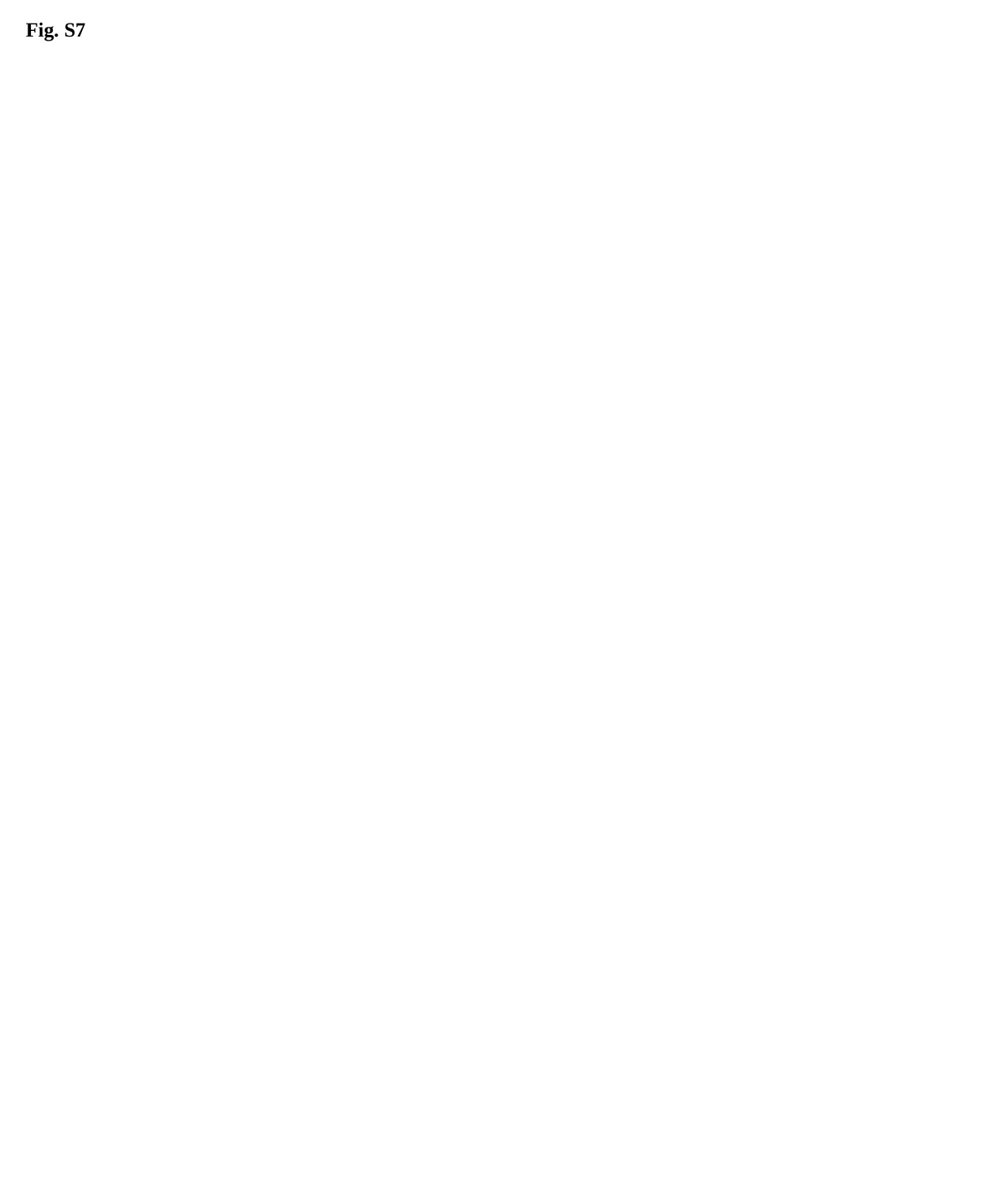

Fig. S7

## Slide 8
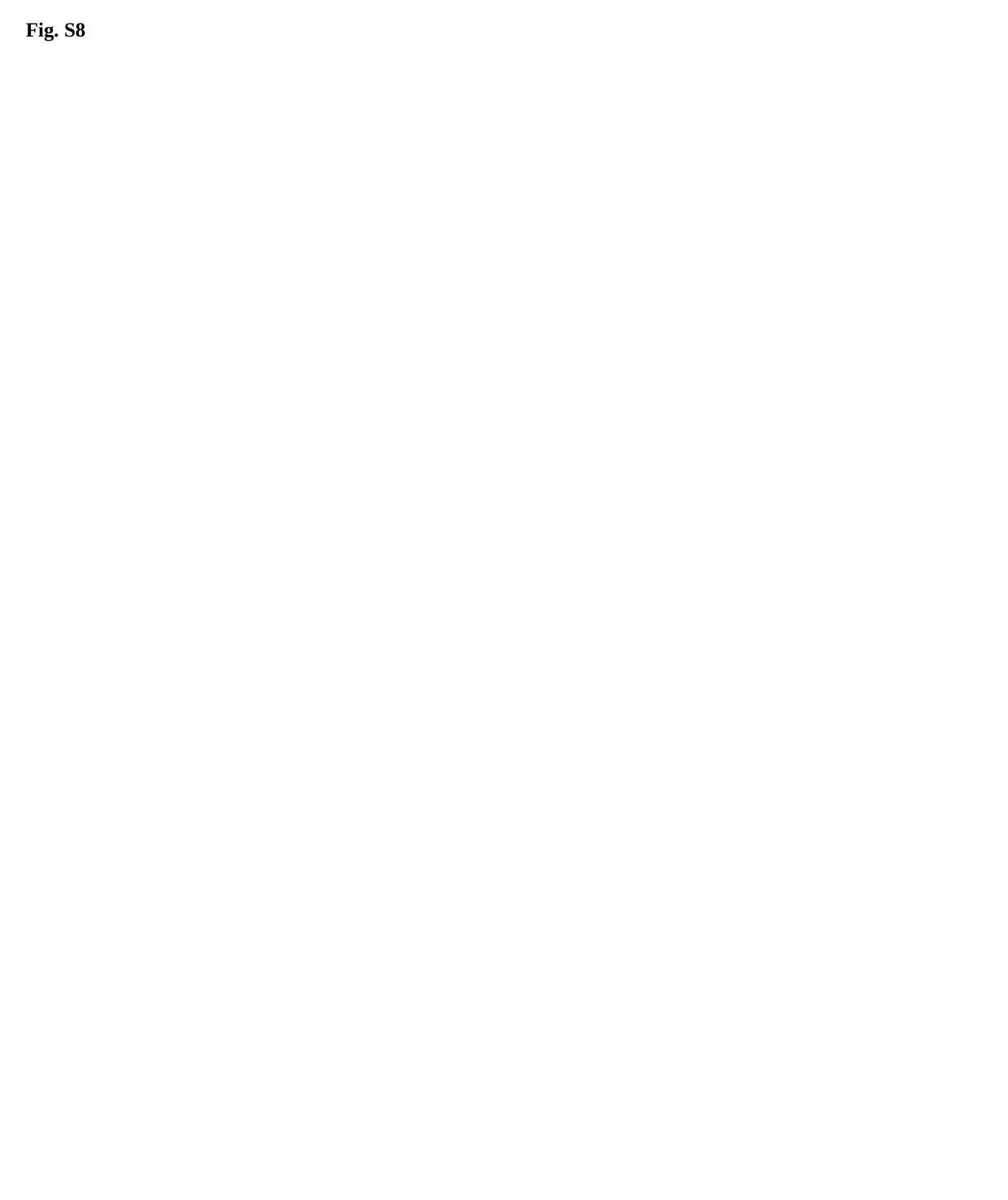

Fig. S8

## Slide 9
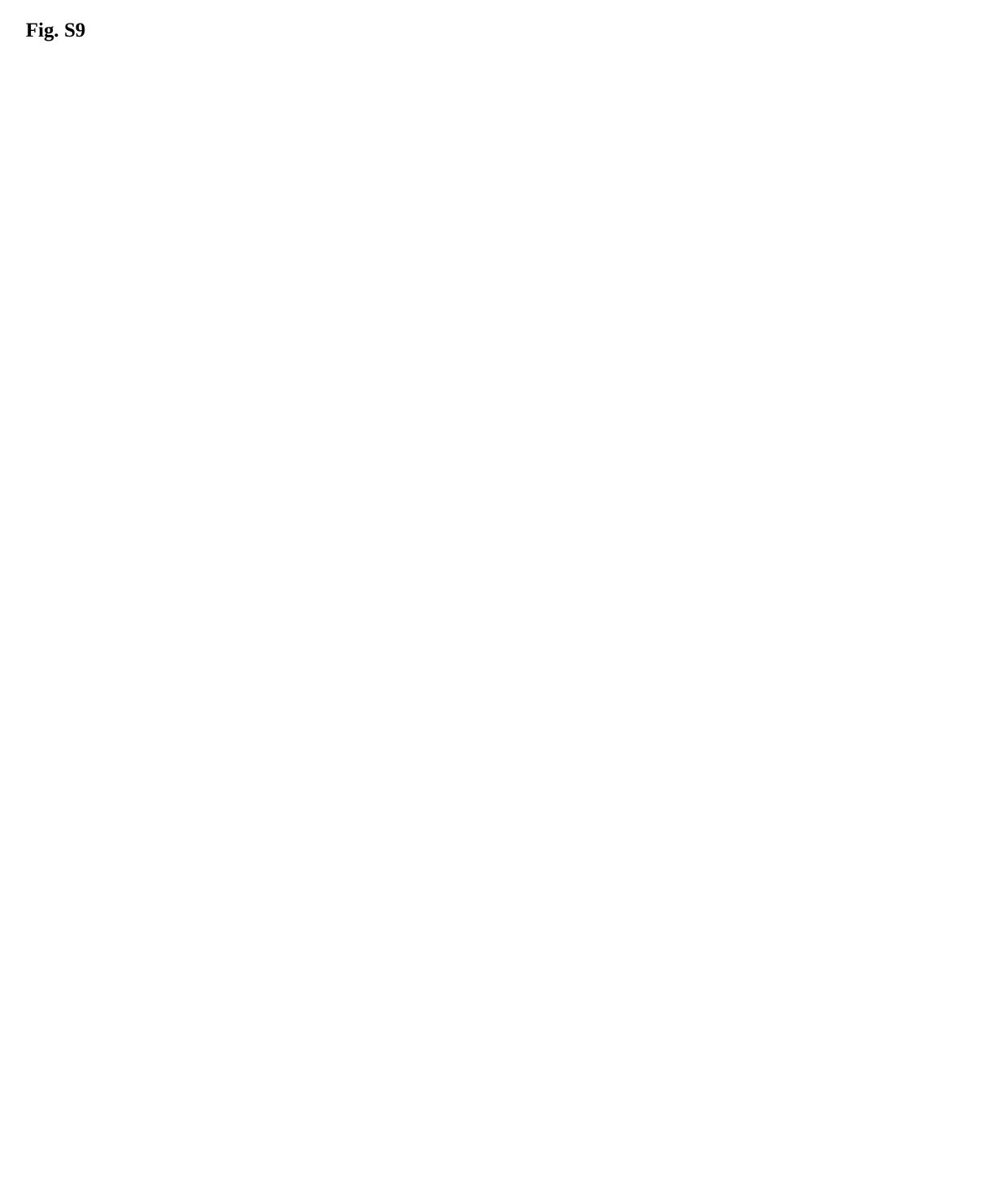

Fig. S9
